# *Rickettsia japonica* and Novel *Rickettsia* Species in Ticks, China

**DOI:** 10.3201/eid2505.171745

**Published:** 2019-05

**Authors:** Xiang-Rong Qin, Hui-Ju Han, Fu-Jun Han, Fu-Ming Zhao, Zhen-Tang Zhang, Zai-Feng Xue, Dong-Qiang Ma, Rui Qi, Min Zhao, Li-Jun Wang, Li Zhao, Hao Yu, Jian-Wei Liu, Xue-Jie Yu

**Affiliations:** Wuhan University, Wuhan, China (X.-R. Qin, H.-J. Han, R. Qi, M. Zhao, L.-J. Wang, J.-W. Liu, X.-J. Yu);; Huangdao District Center for Disease Control and Prevention, Qingdao City, China (F.-J. Han, F.-M. Zhao, Z.-T. Zhang, Z.-F. Xue, D.-Q. Ma);; Shandong University, Jinan, China (L. Zhao); Fudan University, Shanghai, China (H. Yu)

**Keywords:** Rickettsia, *Haemaphysalis longicornis*, vector-borne infections, China, ticks, bacteria

## Abstract

PCR amplification indicated the minimum infection rate of *Rickettsia* spp. was 0.66% in *Haemaphysalis longicornis* ticks collected from Shandong Province, China. Phylogenetic analysis based on the *rrs*, *gltA, ompA*, and *ompB* genes indicated that the ticks carried *R. japonica*, *Candidatus* Rickettsia longicornii, and a novel *Rickettsia* species related to *R. canadensis*.

*Rickettsia* species are gram-negative obligate intracellular bacteria that infect humans and a variety of vertebrates through the bite of arthropod vectors. Hard-body ticks are the primary vector of spotted fever group (SFG) rickettsiae; recently, several emerging and reemerging SFG rickettsiae were found to infect humans (*1*). *Rickettsia japonica* is the pathogenic agent of Japanese spotted fever that has been reported in Japan, South Korea, and Thailand since 1984 (*2*–*4*). Japanese spotted fever is a severe zoonosis and develops abruptly with headache, fever, shaking chills, skin eruptions, tick bite eschars, and malaise (*2*). *R. canadensis* was initially isolated from ticks in Canada; a serologic study indicated the presence of *R. canadensis* antibodies in febrile patients (*5*). The presence of *Rickettsia* species and their distributions in China are not very clear. In this study, we analyzed *Rickettsia* species in *Haemaphysalis longicornis* ticks collected from Shandong Province, China, and found *R. japonica*, *Candidatus* Rickettsia longicornii, and a novel *Rickettsia* species closely related to *R. canadensis* in the ticks.

## The Study

We collected questing ticks by flagging during April–July 2013–2015. We collected them in Jiaonan County (35°35′–36°8′ N and 119°30′–120°11′E), Shandong Province, China. Jiaonan County is located on the Pacific coast of China and has a maritime monsoon-type climate. We identified tick species individually by morphology and confirmed by PCR amplification and DNA sequencing of the 16S rRNA gene of 2 nymphs and 2 adult ticks of each species as described previously (*6*,*7*).

For detection of *Rickettsia* DNA, we pooled ticks according to their developmental stages, with each pool consisting of 20 nymphs or 10 adult ticks. We homogenized them with Tissue Lyser II (QIAGEN, http://www.qiagen.com). We extracted total nucleic acids from the tick suspension using the AllPrep DNA/RNA Mini Kit (QIAGEN).

Initially, in all the tick pools, we amplified nucleic acid preparations with rickettsial universal primers targeting *rrs*, *gltA*, and *ompB* (B1–B4). We further amplified *Rickettsia* clones in the tick pools closely related to *R. japonica* with primers of *ompA*, a SFG rickettsia unique gene. The clones positive with *rrs* and *gltA* gene primers but negative with *ompB* primers (B1–B4) we further amplified with primers Cand-1 to Cand-4, which were designed from the *R. canadensis ompB* gene because the *Rickettsia* clones from these tick pools were closely related to *R. canadensis* on the basis of the *rrs* and *gltA* gene sequences (Table). We used distilled water as a negative control in each run. 

**Table:** Primer sequences and PCR conditions used in study of *Rickettsia* species, China

Target gene	Primer name	Sequence, 5′ → 3′	Amplicon size, bp	Annealing temp, °C	Reference
*rrs*	S1	TGATCCTGGCTCAGAACGAAC	1,486	55	(*8*)
	S2	TAAGGAGGTAATCCAGCCGC			
	S3	AACACATGCAAGTCGRACGG	1,371	55	
	S4	GGCTGCCTCTTGCGTTAGCT			
*gltA*	gltA1	GATTGCTTTACTTACGACCC	1,087	52	(*9*)
	gltA2	TGCATTTCTTTCCATTGTGC			
	gltA3	TATAGACGGTGATAAAGGAATC	667	53	
	gltA4	CAGAACTACCGATTTCTTTAAGC			
*ompB*	B1	ATATGCAGGTATCGGTACT	1,355	56	(*9*)
	B2	CCATATACCGTAAGCTACAT			
	B3	GCAGGTATCGGTACTATAAAC	843	56	
	B4	AATTTACGAAACGATTACTTCCGG			
*ompB*	Cand-1	CCGGACTTTGCGGTGTAGAT	1,136	52	This study
	Cand-2	AAAGCCAGAAGGTGAGGCTG			
	Cand-3	ACCGCACTTGTATCGGTAGT	874	50	
	Cand-4	AAGCAGGTGGTGTAGTCGGA			
*ompA*	Rr190.70p	ATGGCGAATATTTCTCCAAAA	631	50	(*10*)
	Rr190.701n	GTTCCGTTAATGGCAGCATCT			
Tick mitochondrial 16S RNA	Forward	AGTATTTTGACTATACAAAGGTATTG	408	55	(*7*)
	Reverse	GTAGGATTTTAAAAGTTGAACAAACTT			

We performed electrophoresis on the PCR products in 1.2% agarose gels, stained them with ethidium bromide, and visualized them under UV light. DNA bands with the expected size were excised and extracted by Gel Extraction Kit (Omega Bio-tek, https://www.omegabiotek.com). We cloned the purified PCR products into pMD19-T vector (Takara, https://www.takara-bio.com) and engaged Sangon Biotech (Shanghai, China) (https://www.life-biotech.com) to conduct sequencing on both strands. We compared nucleotide sequences with BLAST (http://blast.ncbi.nlm.nih.gov/Blast.cgi) and constructed a phylogenetic tree using the maximum-likelihood method with MEGA version 6.0 (https://www.megasoftware.net). We deposited the *Rickettsia* genes obtained in this study in GenBank under accession nos. MF496152–MF496168 (*rrs*), MF496169–MF496185 (*gltA*), MF496186–MF496199 (*ompB*), and MK102707–MK102720 (*ompA*).

We collected a total of 2,560 *H. longicornis* ticks, 2,080 nymphs and 480 adults. PCR amplification indicated that 14 tick pools were positive with *rrs*, *gltA*, and *ompB* (B1–B4) primers and further positively amplified by PCR with *ompA* primers. In addition, 3 clones were positive with *rrs*, *gltA*, and *ompB* (Cand-1 to Cand-4) primers. The minimum infection rate of *Rickettsia* in the ticks was 0.66% (17/2,560), assuming 1 tick was positive in each positive pool of ticks.

Sequence analysis indicated that 3 clones (J84, J85, and J217) detected from the tick pools were closely related to *R. canadensis*, showing sequence homology of 98.7%–99.1% for *rrs*, 97.8%–98.4% for *gltA* and 94.8%–95.1% for *ompB*. One clone (J244) was highly homologous to *Candidatus* Rickettsia longicornii, showing sequence homology of 99.2% for *rrs*, 100% for *gltA*, and 99.7% for *ompA.* The remaining 13 clones were homologous to each other and to *R. japonica*, showing sequence homology of 99. 2%–100% for *rrs*, 99.1%–100% for *gltA*, 99.3%–99.4% for *ompB*, and 97%–97.3% for *ompA* of a variety strains of *R. japonica* (Appendix Tables 1–4).

Phylogenetic analysis based on the concatenated sequences of *rrs*, *gltA*, *ompB*, and *ompA* showed that *Rickettsia* clones (J84, J85, and J217) were clustered in the same clade with, but distinct from, *R. canadensis*; clone J244 was in the same clade as *Candidatus* Rickettsia longicornii; the remaining 13 clones were in the same clade as *R. japonica*. These results indicated that clones J84, J85, and J217 were a novel *Rickettsia* species; clone 244 was *Candidatus* Rickettsia longicornii; and other clones were *R. japonica* (Figure).

**Figure:**
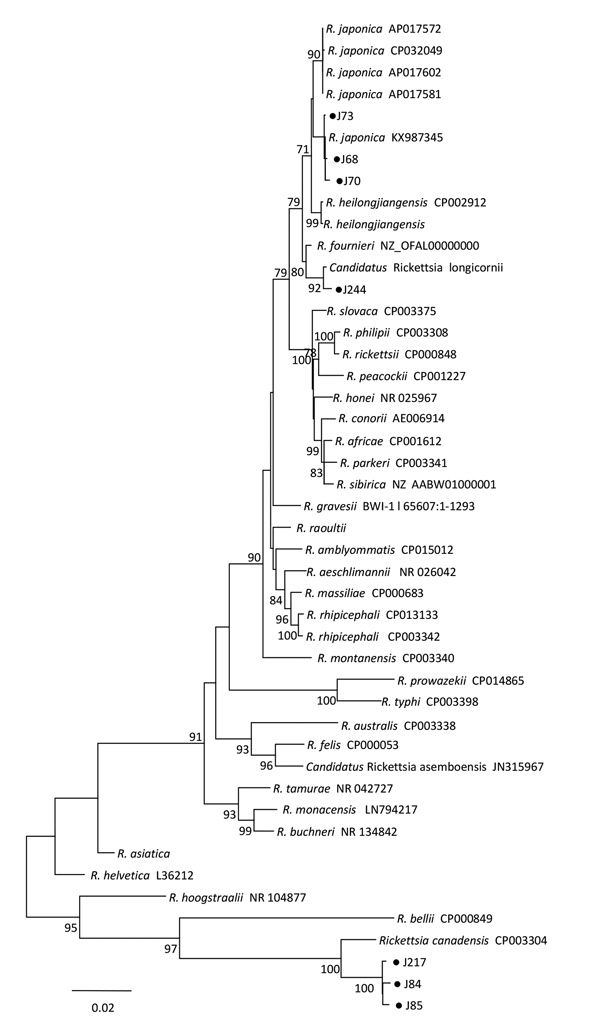
Phylogenetic tree of isolates from study of *Rickettsia* species in China (black dots) and comparison isolates. The tree was generated using the concatenated sequences of *rrs*, *gltA*, *ompB*, and *ompA* of *Rickettsia* species by the maximum-likelihood method in MEGA6 software (http://www.megasoftware.net) with 1,000 replicates for bootstrap testing. Numbers (>70) above or below branches are posterior node probabilities. Dots indicate rickettsial sequences obtained in this study. *Rickettsia* clones J69, J70, and J73 represent 13 similar clones in the phylogenetic analysis. Scale bar indicates nucleotide substitutions per site. The *Rickettsia* species name and complete genome GenBank accession no. appear on each line. For the *Rickettsia* species without complete genome sequences, the GenBank accession nos. in the order of *rrs*, *gltA*, *ompB* and *ompA* are NR_074469, KT899087, and AY280712, AF179362 for *R. heilongjiangensis*; KY474575, KX963389, KU310593, and KX506738 for *R. raoultii*; MG906672, MG906678, and MG906676,0020 for *Candidatus* Rickettsia longicornii; and AF394906, AF394901 and DQ110870 for *R. asiatica*. ppendix. Additional information about *Rickettsia* species in ticks, China.

## Conclusions

In this study, we demonstrated that *H. longicornis* ticks from China were infected with multiple *Rickettsia* species, including *R. japonica*, *Candidatus* Rickettsia longicornii, and a novel *Rickettsia* species. We named the novel species *Candidatus* Rickettsia jiaonani after the sampling site. The exact classification of *Candidatus* Rickettsia jiaonani needs to be further studied by sequencing the whole genomes of the organisms. 

*R. japonica* infection in humans has been reported recently in Anhui Province in central China (*11*), suggesting that *R. japonica* is widely distributed in China and its epidemiology needs to be further investigated. *Candidatus* Rickettsia longicornii was previously detected in *H. longicornis* ticks collected from South Korea (*12*). *Candidatus* Rickettsia jiaonani is closely related to *R. canadensis,* which was first isolated from *H. leporispalustris* ticks removed from rabbits in Ontario, Canada, in 1963 and then from a *H. leporispalustris* tick removed from a black-tailed jackrabbit in California in 1980 (*13*).

*H. longicornis* ticks are native to East Asia, including China, Korea, and Japan, and they were introduced into Oceania, including Australia, New Zealand, Fiji, and Hawaii, through cattle importation (*6*). Recently, this tick species was found in 8 states in the eastern United States (*14*). This study and previous studies demonstrated that *H. longicornis* ticks carry *R. japonica*, *Candidatus* Rickettsia longicornii, *Candidatus* Rickettsia jiaonani, *Anaplasma phagocytophilum*, *Ehrlichia*, and severe fever with thrombocytopenia syndrome virus (*12**,**15*). These pathogens need to be monitored in countries in East Asia in which the *H. longicornis* tick is native and in the countries that this tick species has invaded.

Appendix Additional information about *Rickettsia* japonica and novel *Rickettsia* species in ticks, China.
